# Self-imposed pressure or joyful learning: emotions of Chinese as a foreign language learners in feedback on academic writing

**DOI:** 10.3389/fpsyg.2024.1463488

**Published:** 2025-01-07

**Authors:** Ruoxi Liu, Ping Xin

**Affiliations:** School of Chinese as a Second Language, Faculty of Humanities, Peking University, Beijing, China

**Keywords:** positive psychology, CFL academic writing, feedback, academic emotion, emotion regulation strategies

## Abstract

Although writing feedback is widely believed to elicit a range of emotions, studies on the emotional experiences of L2 students with this teaching and learning tool, as well as their regulation strategies, remain largely underexplored. Drawing on the analytical framework of academic emotions from the perspective of positive psychology, this study examines two Chinese as foreign language (CFL) students’ emotional reactions to their teacher’s oral and written feedback and their emotion regulation strategies. The main data includes interviews, retrospective oral reports, students’ reflection journals, academic writings, and teacher feedback. The study found that feedback aroused students’ academic achievement emotions, cognitive emotions, and social emotions across various dimensions of valence and activation. Over the course of three feedback processes within one semester, the two learners’ emotions gradually became neutral or positive. They effectively employed emotion-oriented, appraisal-oriented, and situation-oriented strategies to manage negative emotions and adapt to feedback. The findings suggest that paying attention to the intrinsic values of feedback may help learners experience more positive academic emotions, while paying too much attention to its extrinsic values may lead to negative emotions.

## Introduction

The process of learning academic writing is filled with difficulties and challenges, especially for novice second-language writers who inevitably experience a range of complex emotions (Han and Hyland, 2019). Feedback, as a critical tool in writing instruction, refers to written or oral comments and revision suggestions on the language or content of a learner’s writing. It plays a pivotal role in shaping the quality of second-language learners’ writing output (Kepner, 1991). Learners are the primary agents of feedback information processing (Winstone et al., 2022), and the effectiveness of this processing directly impacts their writing abilities.

As an essential part of feedback and academic writing learning (Hyland and Hyland, 2006), emotional experiences significantly affect learners’ L2 learning achievements, feedback engagement, and the effectiveness of their processing (Burić et al., 2016). Many teachers advocate for providing positive feedback while selectively providing negative feedback to reduce learners’ negative emotions (Han and Hyland, 2019), enhance their learning behavior and sense of agency, and improve their academic achievement.

In recent years, positive psychology has led to the emotional turn in second language acquisition, and more and more scholars have called for attention to both individual learning outcomes and their emotional experiences (Dewaele and Li, 2020). However, research on the emotional experiences of second language learners in response to writing feedback is still insufficient; most existing studies focus on single writing feedback (Han and Xu, 2020; Geng and Yu, 2024), so it is impossible to observe the dynamic development of an individual’s emotional experiences over a period of time after several feedback sessions and whether emotions can be effectively improved through regulatory strategies. In addition, previous studies have mostly assumed that learners with lower language proficiency and writing ability are more likely to receive more feedback, resulting in more negative emotions with higher activation levels being aroused (Hyland, 1998; Jiang and Dewaele, 2019). The specific relationship between learners’ academic achievement and emotional experiences needs to be analyzed in more detail. Based on this, to advance our understanding of the emotional dimension of feedback, this study investigated the dynamic changes in the emotional experiences and regulation strategies of two undergraduate CFL students during a one-semester Chinese academic writing course evoked by three feedback sessions.

## Literature review

Emotions typically include both trait emotions (habitual and recurring) and state emotions (“momentary occurrences within a given situation at a specific point in time”) (Pekrun, 2006, p. 317). This study uses a qualitative approach to explore the emotional experiences of individual CFL students in response to feedback from a cognitive perspective. Therefore, we focus primarily on state emotions, which are understood as subjective academic emotions that are triggered by the moment when CFL students receive and process feedback.

### Academic emotions and emotion regulation

Academic emotions refer to a series of emotions experienced by learners during academic activities. They are subjective psychological and physiological states that are directly related to academic learning, classroom teaching, and academic achievement (Pekrun et al., 2002; Imai, 2010). Among several theoretical approaches to the study of emotions (Gross, 1998; Han and Gao, 2024), Pekrun and his colleagues have provided a useful analytical framework for the study of academic emotions (Pekrun, 2006; Pekrun and Linnenbrink-Garcia, 2012). This framework considers multiple dimensions of emotions and is an analytical framework that has been widely used and recognized in the current field of academic writing to study L2 learners’ emotions (e.g., Han and Hyland, 2019; Han and Xu, 2020; Geng and Yu, 2024). Pekrun (2006) research on academic emotions has conceptualized emotions as varying along two dimensions, i.e., valence and activation, and having different object foci. Valence refers to the positive–negative dimension of emotion, whereas activation refers to the degree of emotional arousal, which can be divided into activating and deactivating (Pekrun et al., 2002). On this basis, academic emotions are divided into positive activating emotions, positive deactivating emotions, negative activating emotions, and negative deactivating emotions. Although this framework has been widely applied to explore learners’ emotional experiences, its establishment mainly relies on questionnaires. A single research method may not be able to comprehensively and in-depth reveal individuals’ emotional changes, nor can it disclose their differences based on individual experiences, personalities, etc. Moreover, the framework was mostly developed based on the Western cultural background, while learners from different cultural backgrounds may have different understandings and experiences of emotions. Therefore, the analytical frameworks of Pekrun (2006) and Pekrun and Linnenbrink-Garcia (2012) still need to be further verified and explored in different situations, cultural backgrounds, and individual emotional experiences of learners.

It is generally believed that positive emotions can expand learners’ thought-action repertoire in the short term, which is conducive to building long-term cognitive, motivational, and social resources (Fredrickson, 2001), whereas excessive negative emotions may hinder their learning investment (Li et al., 2023; Han et al., 2024). However, academic emotions have the same valence but different activations that have different effects on academic achievement (Pekrun et al., 2002). For example, negative activating emotions (e.g., anxiety) may produce task-irrelevant thinking and undermine the students’ intrinsic motivation, whereas negative deactivating emotions (e.g., hopelessness) could impair students’ performance by undermining both intrinsic and extrinsic motivation, learning engagement, and promoting superficial information processing (Pekrun, 2006; Geng and Yu, 2024). In addition to valence and activation, Pekrun and Linnenbrink-Garcia (2012, p. 262) and Han and Hyland (2019) also grouped academic emotions according to their object focus. Table 1 categorizes academic emotions by object focus in feedback situations.

**Table 1:** Academic emotions are categorized by object focus in feedback situations (Han and Hyland, 2019; Pekrun and Linnenbrink-Garcia, 2012).

Category	Definition	Definition in feedback situations
Achievement emotions		
-Activity emotions	Emotions are aroused when engaging in learning activities.	Emotions are aroused by processing and using feedback to improve the writing.
-Prospective outcome emotions	Emotions aroused by future expected outcomes.	Emotions are aroused by (a) the expected accuracy of the writing before receiving feedback and (b) the expected accuracy of the revised writing during revision.
-Retrospective outcome emotions	Emotions aroused by past task outcomes.	Emotions are aroused by (a) the written accuracy of the previous writing(s) after receiving feedback and (b) the written accuracy of the revised writing after revision.
Epistemic emotions	Emotions are aroused by cognitive processing during the task.	Emotions are aroused by the cognitive processing of feedback.
Social emotions	Emotions aroused by other persons.	Emotions are aroused by other persons, e.g., teachers and classmates, in feedback situations.
Topic emotions	Emotions aroused by the contents of learning material (e.g., empathy with the characters in a novel).	Emotions pertaining to the topic and content of the writing task.

Regarding academic emotional experiences, Pekrun (2006) also proposed the control-value theory, which argues that emotions are mainly influenced by two factors: control appraisal and value appraisal. Control appraisal refers to learners’ perceptions of their ability to control past, present, and future academic activities or outcomes. Value appraisal involves learners’ judgments about the significance of academic activities or outcomes, which can be divided into intrinsic values and extrinsic values.

Intrinsic values refer to the subjective importance learners attach to an activity itself, regardless of its outcomes. For example, students who enjoy memorizing Chinese vocabulary and find it meaningful may value the activity even if it does not directly improve their Chinese learning performance. In contrast, extrinsic values view the activity as a means to achieve a specific outcome. For example, students might focus on improving their Chinese writing skills because it directly contributes to better scores on preparatory exams and increases their chances of entering undergraduate studies (Pekrun, 2006). Despite the importance of these factors, studies examining the influence of control and value appraisals on the emotional experiences induced by feedback remain scarce in current research (Han and Hyland, 2019).

Many studies have confirmed that individuals can regulate and cognitively process emotional experiences (Mahfoodh, 2017; Pitt and Norton, 2017; Geng and Yu, 2024). Academic emotion regulation is the process by which individuals influence the emotions they arouse when emotions occur and the expression of emotional experiences in a learning environment (Gross, 1998). Inspired by control value theory, Pekrun (2006) further proposed an analytical framework for academic emotion regulation strategies that include four emotion regulation strategies: emotion-oriented regulation, appraisal-oriented regulation, problem-oriented regulation, and situation-oriented regulation. Definitions of the emotion regulation strategies are given in Table 2. This framework is currently widely used to study emotion regulation in the field of L2 academic writing (Han and Xu, 2020). Based on this framework, this study will further explore CFL students’ emotion regulation strategies in feedback and explore whether these strategies can effectively improve their negative emotions.

**Table 2:** Academic emotion regulation strategies (Pekrun, 2006).

Strategies	Definitions
Emotion-oriented regulation	Regulate academic emotions directly (e.g., focusing attention on the emotion or distracting it away, using relaxation techniques, or taking drugs).
Appraisal-oriented regulation	Addresses the control and value antecedents of emotions (e.g., restructuring expectancies and attributions).
Problem-oriented regulation	Focuses on improving academic learning and achievement underlying perceived control (e.g., acquiring study skills).
Situation-oriented regulation	Attempt to change situational circumstances defining controllability and values (e.g., by asking for a reduction of task demands or by dropping out of a course).

### Emotional experiences and emotion regulation in feedback on L2 academic writing

In the field of academic writing, previous studies have found that novice writers are more likely to experience multiple emotions, such as anxiety and pleasure, when receiving feedback (Lei and Hu, 2019; Yu and Jiang, 2022; Gao and Yang, 2023) or ultimately promote skepticism about the review (Kong and Teng, 2023), which affects their comprehension speed and feedback acceptance (Mahfoodh, 2017). Different types of feedback lead to different emotional experiences for learners. For example, some feedback content is highly critical, and the expression is direct and face-threatening, which is likely to evoke strong negative emotions (Madhu and Hu, 2021). Some researchers believe that feedback can easily arouse negative emotions and thereby undermine learners’ enthusiasm (Truscott, 1996). However, the study of emotions in L2 academic writing from the perspective of positive psychology takes a holistic view of the integration of positive and negative emotions (MacIntyre et al., 2019) and has reached different research conclusions. For example, some learners experience gratitude, disappointment, happiness, embarrassment (Han and Xu, 2020), frustration (Zheng and Yu, 2018), relief, and excitement after receiving feedback (Han and Hyland, 2015; Mahfoodh, 2017), revealing the complexity and situational and dynamic nature of emotions in L2 writing. The study by Han and Hyland (2019) was among the earlier studies exploring emotional experiences in L2 feedback, and used Pekrun (2006) and Pekrun and Linnenbrink-Garcia (2012) emotional analysis framework to explore English second language learners’ emotional experiences in feedback, providing references and examples for subsequent research on L2 learners’ emotional experiences elicited by feedback. The study used qualitative research methods supplemented by classroom observation recordings and students’ facial expressions for triangulation, revealing two learners’ emotional experiences, such as calmness, guilt, and nervousness, in response to written corrective feedback (Han and Hyland, 2019). Geng and Shulin (2024) study showed that learners had 65 discrete emotions after receiving feedback, the most common of which were negative emotions, but these negative emotions did not discourage them because they actively sought external resources to improve their revisions. It can be seen that the previous view that feedback always elicits negative emotions may not be true (Truscott, 1996; McMartin-Miller, 2014) and perhaps does not reflect the actual experience of second language learners.

Some studies have explored learners’ emotion regulation after receiving feedback and found that learners can promote positive emotions through a variety of strategies (Liu and Yu, 2022). Teachers can also use psychological interventions to create a positive language learning environment (MacIntyre et al., 2019) and guide learners to develop learning emotional intelligence. Most studies are based on large-scale quantitative analyses. For example, Han et al. (2024) conducted a questionnaire survey on 363 postgraduate students and found that they can adopt strategies such as emotion-oriented regulation and situation-oriented regulation to regulate emotions; Teng and Ma (2024) demonstrated the importance of self-perceived motivation and confidence in regulating students’ emotions and proposed that these findings should be used throughout the feedback process to facilitate students’ seeking, generating, processing, and using feedback. Through focused writing and semi-structured interviews with middle school students, Gu et al. (2022) found that seeking social interaction, developing competence, and cognitive reappraisal were the most commonly used strategies by students. At the same time, specific academic emotion regulation strategies are related to the purpose of emotion regulation. For example, students most often use the strategy of developing competence when they want to learn from their peers’ essays.

In general, current research on L2 writing feedback focuses on its role and different methods. The little attention paid to learners’ emotions mostly revolves around the changes in emotional experiences before and after a particular feedback (Ellis, 2010) and has not extended the observation to dynamic situations such as longer periods of time and multiple writing sessions. Furthermore, academic emotions and emotion regulation have generally been studied in English L2 writing contexts or in the context of master’s or Ph.D. theses (e.g., Mirka and Kirsi, 2019; Geng and Yu 2024), with little attention paid to Chinese L2 undergraduate students. As these learners are new to academic writing and studying in a foreign culture, their emotional experiences are richer and more complex than those of learners who study L2 in their home countries and those who have more writing experience, and thus merit in-depth exploration through qualitative means such as case studies, as they can offer unique insights into the complex interplay between emotions and learning in a cross-cultural educational environment.

In addition, previous studies have found that an individual’s emotional experience is not only influenced by the feedback itself but also generated by the interaction of many factors, such as individual and social context (Jin and Zhang, 2018; Jiang and Dewaele, 2019). Regrettably, no research to date has delved into the specific contextual factors that shape these emotional experiences. To enhance our understanding of how CFL students navigate their emotions and employ emotion regulation strategies in response to feedback and to extract implications with far-reaching instructional significance and applicability across diverse educational settings, the current study endeavored to address the following two research questions:

1. How do CFL students’ emotional experiences change throughout the teacher’s three feedback sessions and each time’s revision process?
2. What regulation strategies did CFL students use to self-regulate the academic emotions aroused by the teacher’s feedback?

## Research methods

### Research context

The data for this study come from a 16-week academic Chinese writing course for international undergraduates offered by a Chinese university in the spring semester of 2023. The course was taught in Chinese and aimed to cultivate students’ awareness of genre and norms in academic writing, which could prepare them for future academic research and dissertation writing. There are three summary writing tasks in one semester (in the third, eighth, and twelfth weeks, respectively). The students are required to screen and extract the key points of the original text based on reading and understanding an academic paper and write a summary in their own language. The instructor is a native Chinese-speaking teacher from China, Audrey (all names are pseudonyms), who majored in Chinese language and literature in her undergraduate, master’s, and doctoral degrees and has 25 years of experience in teaching Chinese L2 academic writing. Before assigning the writing task, the teacher (Audrey) had already taught the methods of summary writing in class. The students completed the writing in class, and the teacher (Audrey) gave each student written feedback and oral feedback in class.

### Participants

This study aimed to select students so that there would be individual differences among the participants in terms of writing experiences and emotional experiences. We used maximum purposive sampling, and two students, Yuki and Sala, agreed to participate. The participants are from different countries around the world. Due to the uneven development of Chinese language proficiency, writing experiences, and beliefs and attitudes toward academic writing feedback, their emotional experiences of academic writing feedback are also different. As the researchers are involved in teaching the course, they are very familiar with the participants. We recruited the participants through the first researcher, and they will provide information about their feedback and academic writing experiences. The participants were purposively selected based on three specific criteria: (a) they both had a strong desire to participate in the research and were willing to share their experiences and emotions in academic writing, which provided feasibility for the smooth progress of the research; (b) they both needed to write their dissertations in Chinese when they graduated in the future; (c) they had different HSK (Chinese Proficiency Test) level 6 writing scores, previous academic writing performance, first summary writing scores (Yuki was a high-level writer and Sala was a low-level writer), the feedback they received, and their academic emotions showed an undeniable contrast. The two cases can reflect the students’ emotional experiences and regulation in writing feedback. Yuki’s and Sala’s backgrounds are given in Table 3.

**Table 3:** The CFL students’ background information.

	Yuki	Sala
Gender	Female	Female
First language	Japanese	Thai
Major	Journalism and Communication	Journalism and Communication
Year of college	First-year	First-year
Writing score of HSK-6	75	60
Summary writing score 1	84	79
Summary writing score 2	87	80
Summary writing score 3	89	88

It is worth noting that only two learners were selected as subjects in this study. The purpose of doing so was to choose individuals with specific characteristics or experiences and thoroughly explore their detailed experiences and reactions in specific situations. This helps to focus on the research questions and avoid the situation where the information becomes too scattered due to an overly large sample, making it impossible to deeply analyze the relationships between key factors. In addition, diverse data collection methods were used for these two students in this study to obtain comprehensive and in-depth data, thus compensating for the deficiency of the small sample size.

### Data collection

The emotional reactions to the teacher’s feedback of student participants were mainly investigated through interviews, retrospective oral reports, audio recordings, and class observation notes, and the teacher’s written feedback was also collected as Supplementary Data. That is, all the methods mentioned above are used to answer Question 1. The emotion regulation strategies were analyzed mainly through interviews and retrospective oral reports. Thus, the two methods are used to answer Question 2.

- Interviews: Three formal, semi-structured interviews (see Supplementary Appendix 1 for the interview guidelines) and multiple informal interviews. The first interview was conducted at the beginning of the semester and lasted approximately 40 min, mainly to explore learners’ personal information, experiences of academic writing and dealing with feedback, and beliefs about academic writing and feedback. The next two interviews focused mainly on learners’ emotional experiences of each piece of writing feedback and future academic writing and regulation strategies. In the opening part, the first researcher explained the purpose and procedure of the study. In the questioning part, the participants were encouraged to give detailed insights into their emotional reactions to the feedback and how they regulated their emotions to proceed with the writing. The semi-structured interviews were conducted within 48 h of the learners receiving the first (F1), second (F2), and third (F3) feedback, respectively, and each interview lasted approximately 60–90 min. The informal interviews were mainly based on daily communication via WeChat and face-to-face communication, with the aim of supplementing the information not obtained in the formal interviews and tracking the real state and inner experience of the participants in their learning life.
- Retrospective oral report: students conducted a 5–10 min retrospective oral report within 24 h after receiving the three feedback. Students were asked to report on their emotional experiences and regulation strategies before and after receiving feedback, and the researcher recorded their responses to the feedback. To stimulate recall, participants were able to review the written text, the teacher’s written feedback, and the error log to recall their experiences and attitudes toward feedback.
- Class observation notes: One of the researchers was a teaching assistant for the Chinese academic writing course, who was able to observe the class in depth and know the language level and writing ability of the participants.
- The teacher’s written feedback: Audrey gave feedback and scores to each participant’s summary writing text. This data can reflect the participants’ writing ability and the type of feedback given by the teacher.

We also took the following measures to keep the participants’ information confidential: During data collection, each participant was anonymized, and pseudonyms were used to label all content, such as interview records and reflection logs; in the data processing stage, the data was stored in password-protected software, and only the researcher could directly access the original data; in the data use stage, the research members signed a confidentiality agreement, promising not to disclose the data content; in the paper writing stage, the identity information of the participants was thoroughly anonymized to ensure that it could not be traced back to specific individuals.

In addition, in order to prevent adverse emotional reactions of the subjects during interviews and self-reports, we also took a series of relevant measures. First, when recruiting the subjects, we informed them of the possible emotional challenges involved in the research process, such as the adverse emotions that might be triggered by recalling difficult experiences in the learning process and facing critical feedback. We ensured that the participants voluntarily participated in the research with full knowledge and provided them with the right to withdraw at any time. Second, the researcher (the first author of this article) familiarized himself/herself in advance with the types of possible adverse emotional reactions and the corresponding coping methods and provided the subjects with information about the school’s mental health center for them to seek help. Third, during the research activities, such as interviews and classroom observations, we closely monitored the emotional states of the participants. Once we found that the participants had emotional fluctuations, we immediately suspended the research activities and, at the same time, gave the participants the opportunity to express their emotions. We used active listening and encouragement techniques. Finally, we conducted follow-up visits to the participants to understand their emotional recovery situations and reminded them that they could continue to seek help if they still had emotional problems.

### Data analysis

#### Analysis of the teacher’s written feedback

Writing text analysis is mainly concerned with defining the amount of feedback given by the teacher. According to Hyland (1998), a single feedback point is defined as each opinion expressed on a particular aspect of the article, including each comment and revision. This study refers to the classification of Ellis (2009) to classify feedback points from two dimensions: one is non-corrective feedback, which refers to the teacher’s comments on the writing (Ferris, 1997); the other is corrective feedback, including direct feedback (correcting errors directly) and metalinguistic feedback (explaining the metalinguistic rules violated by the error). The specific content is shown in Table 4.

**Table 4:** The CFL students’ feedback information.

Name		Non-corrective feedback (comments)	The teacher’s written feedback
Yuki	F1	Ideological content comments (negative) 3 Overall improvement suggestions 1	Direct feedback 9 Indirect feedback 0 Metalinguistic feedback 3
	F2	Organizational structure comments (positive) 1 Language usage (negative) 1	Direct feedback 10 Indirect feedback 0 Metalinguistic feedback 1
	F3	Language usage comments (positive) 1 Ideological content comments (positive) 1 Overall improvement suggestions 1	Direct feedback 9 Indirect feedback 0 Metalinguistic feedback 2
Sala	F1	Ideological content comments (positive) 1 Overall improvement suggestions 1	Direct feedback 15 Indirect feedback 0 Metalinguistic feedback 2
	F2	Ideological content comments (positive) 1 Organizational structure comments (negative) 1 Overall improvement suggestions 1	Direct feedback 15 Indirect feedback 0 Metalinguistic feedback 6
	F3	Language usage comments (negative) 1 Ideological content comments (positive)1 Organizational structure comments (negative) 1 Overall improvement suggestions 1	Direct feedback 16 Indirect feedback 0 Metalinguistic feedback 5

#### Analysis of other data

Data analysis adopts the qualitative content analysis method (Miles et al., 2013; Han and Xu, 2020). Individual case files are established for the data, and qualitative analysis is carried out according to the steps from within - individual cases to cross-individual cases (Han and Hyland, 2019). Data were reviewed by thematic analysis and the constant comparative method of analysis, in which the data were systematically analyzed through a three-stage process of first and second-cycle coding for data condensation (Miles et al., 2020). The coding process was as follows: First, the within-case analysis stage. The recording was transcribed immediately after each interview, and data analysis began immediately after review and approval by the participants. Micro-analysis of the data was carried out after the first written feedback from the first participant. The data were read repeatedly. Using the academic emotion classification framework (Pekrun, 2006; Pekrun and Linnenbrink-Garcia, 2012; Han and Hyland, 2019) and the emotion regulation strategy classification framework (Pekrun, 2006), relevant data fragments related to the research questions were cyclically coded and classified, and the analysis framework was adjusted according to the data. Emotional experiences and regulation strategies were mainly identified using vocabulary from student interviews, self-reports, and classroom observation notes. Then, a preliminary coding list was produced, including emotional experiences and regulation strategies. Second, the data of the second participant were analyzed, and the coding list was improved. Thirdly, in the cross-case horizontal comparison stage, case narratives are completed by refining themes and taking an inductive approach. The coding lists of the two participants were compared to select, organize, and merge important concepts and dimensions. For example, ‘immediate emotions’ and ‘emotions after reading feedback’ were grouped into one category and coded as ‘emotions during academic work.’ The frameworks (Pekrun, 2006; Pekrun and Linnenbrink-Garcia, 2012; Han and Hyland, 2019) were adapted according to the specific content of the data:

(a) Five discrete emotions were added to Pekrun and Linnenbrink-Garcia (2012) taxonomy: conflict, the feeling of being torn because you do not know exactly which way is better for you; novelty, the feeling of being strange and new because of something you have never seen or experienced before; achievability, the feeling when desires and reality are in balance; relief, the positive feeling induced by the fact that the expected failure did not actually occur; anticipation, the feeling of longing and yearning for something unknown in the future. Definitions of all discrete emotions are given in Supplementary Appendix 2. (b) Through a detailed review of the interview, oral report, and classroom observation of the present study. It was found that when facing academic writing feedback, the participants more often adopted other types of strategies (such as emotion-oriented regulation, appraisal-oriented regulation, and situation-oriented regulation strategies) to deal with emotional problems and did not clearly exhibit the behavior pattern of directly solving the problem itself (i.e., “problem-oriented strategy”), such as seeking additional learning resources to specifically solve the problems exposed in writing or changing the learning method to avoid similar problems from recurring. Therefore, it was removed from the classifications.

Except for identifying discrete emotions, informed by research on academic emotions, a dimensional approach was also taken to identify the object focus, valence, and activation of emotions. The discrete emotions were categorized by object focus following Pekrun and Linnenbrink-Garcia (2012) classifications (see Table 1). This study did not find that the feedback aroused the learners’ “topic emotions” (emotions related to the content of the writing task). Therefore, it was removed from the analytical framework. Pekrun (2006) assigns different valences and activations to discrete emotions, e.g., ‘anxiety’ belongs to the negative-activating dimension and ‘excitement’ to the positive-activating dimension. However, based on the interviews and participants’ reports, we found that the degree of activation of the same emotion varied. For example, Yuki felt anxious before receiving the three feedback sessions, but she thought that the activation of anxiety at the first feedback was the strongest and then gradually decreased. Therefore, in this study, the activation of these three anxiety emotions was coded as activating, neural, and deactivating, respectively.

In addition, to avoid the influence of the participants’ L2 proficiency on the expression of meaning, this study used a triangulation method for the assessment of emotions and strategies. Except for the analysis of interviews and self-reports, the following data were also included: phonetic details and gestures during interviews, self-reports, students’ reflection logs, and the researcher’s classroom observations. Finally, the above data were archived by case, followed by horizontal comparisons across cases, and the case narratives were completed by refining themes. Two researchers independently coded the above data and initially achieved 83% inter-coder agreement. The researchers then discussed the different codes of discrete emotions, valence, activation, and emotion regulation in order to resolve the differences. Inter-coder reliability eventually reached 96%. The coding schemes for CFL students’ emotional reactions to feedback are shown in Table 5, and the academic emotions categorized by object focus and examples are shown in Table 6.

**Table 5:** Coding schemes of emotional responses to feedback.

Codes of emotional responses to feedback	
Discrete emotions	Conflict, anxiety, hopelessness, guilt, novelty, confusion, tranquility, achievability, gladness, trust, hope, expectancy, gratitude, relief, curiosity, and satisfaction
Object focus	Achievement emotions (including prospective outcome emotions, retrospective outcome emotions, and activity emotions), epistemic emotions, and social emotions
Valence	Positive, neutral, and negative
Activation	Activating, neutral, and deactivating

**Table 6:** Academic emotions categorized by object focus and examples in feedback situations.

Category	Discrete emotions	Examples
Achievement emotions		
-Activity emotions	Novelty, confusion, anxiety, gladness, relief, achievability, tranquility	Yuki: Maybe I am a bit unsophisticated. It was the first time I saw the revisions on the computer (oral report) Sala: I was very happy after reading the comments, and I found that the teacher praised me (interview)
-Prospective outcome emotions	Conflict, novelty, expectancy, anxiety, tranquility, hope	Yuki: Before receiving the feedback, I felt conflicted (reflection log); Sala: This time should be an improvement over the last time. I was quite tranquil (reflection log)
-Retrospective outcome emotions	Confusion, guilt, tranquility	Yuki: I feel guilty, as always, for wasting the teacher’s time (oral report)
Cognitive emotions	Hopelessness, guilt, curiosity	Yuki: I have tried my best, but it seems that my ability is still not enough (interview)
Social emotions	Tranquility, gratitude, anxiety, trust, relief	Yuki: I am grateful to the teacher. She spent a lot of time revising (reflection log) Sala: I am worried that my Chinese is not good enough. I am really anxious to meet the teacher (oral report)

## Findings

### Yuki: academic emotions and regulation strategies under self-imposed pressure

Yuki comes from a bilingual Chinese–Japanese family in Japan. She attended high school in northeast China. She was very concerned about her GPA and planned to study for a master’s degree, but she did not know how to express herself in an academic genre. Yuki believed that summary writing and feedback could improve academic writing skills, but the skills gained from feedback on a specific piece of writing could not be transferred to other papers. Yuki’s overall emotions aroused by feedback were “strongly changing and up and down” (the third oral report) and constantly evolving between negative and positive, even though her scores on three pieces of writing were among the best in the class. Based on the oral report and the interviews, the researcher summarized the key times of emotional change and the changes in Yuki’s academic emotions (see Table 7).

**Table 7:** Yuki’s academic emotions to three feedback sessions and regulation strategies.

		Before receiving	Feedback receiving	Feedback reading	Oral feedback in class	After revision
F1	Academic emotions	Conflict anxiety	Hopelessness guilt	Novelty confusion anxiety	Tranquility gratitude	Confusion guilt
	Valence-Activation	Negative-activating Negative-activating	Negative-activating Negative-activating	Positive-neutral Negative-deactivating Negative-activating	Neutral-neutral Positive-activating	Negative-Deactivating Negative-activating
	Strategies	Emotion-oriented regulation	Situation-oriented regulation			
F2	Academic emotions	Anxiety	Relief achievability	Confusion	Glad trust	Tranquility
	Valence-Activation	Negative-neutral	Positive-neutral Positive-neutral	Negative-deactivating	Positive-neutral Positive-neutral	Neutral-neutral
F3	Academic emotions	Anxiety	Tranquility	Confusion	Glad	Hope
	Valence-Activation	Negative-deactivating	Neutral-activating	Negative-neutral	Positive-neutral	Positive-neutral

#### A rough start: walking alone in anxiety and guilt in the first feedback session

The most common emotions Yuki experienced when she first received feedback on her academic writing were guilt and anxiety. After finishing her first piece of writing, Yuki felt anxious and conflicted as she waited for the feedback email. “I’m always worried about the grade. I hope the teacher will revise it more so that I can learn a lot, but the score will be low” (first oral report). So, she quickly looked at the score as soon as she received the email.

[First interview].

“My first reaction was, ‘It’s over.’ I did not even get 85. I tried my best, but it seems that I’m still not good enough. I may have to catch up on this GPA in another course. Then came that familiar feeling of guilt.”

During the interview, she mentioned the emotions of “hopelessness and guilt,” even though it had been 36 h since she had received the feedback, and her eyes were red, showing that she was in a negative-activating state. After looking at the score, Yuki prepared to read the feedback carefully. What caught her eye was the revision that Audrey had made using the ‘word revision mode.’ It felt very novel. However, much of the content of the feedback made her feel confused and anxious:

[First interview].

“I’m not used to the ‘Word revision mode.’ The teacher also said that a sentence was repeated, but I did not really notice it. To be honest, I do not know if it was because I was too anxious and could not think, which led to me not being able to understand these things after they were revised.”

When talking about emotion regulation that could be used to improve these emotions, Yuki stated that she “refused to regulate” and that “living in anxiety is especially good because it can motivate me to study harder. Maybe only grades can make me really happy” (oral reports).

The teacher then gave oral feedback to the class on the common writing problems, which resolved most of Yuki’s confusion. Audrey praised the students’ efforts, smiled, and nodded to encourage everyone. Yuki also had social emotions such as gratitude. “If the teacher did not tell me some problems, I might never know them in my life” (first interview). Yuki aroused social emotions (gratitude) to “hedge” negative emotions through emotion-oriented regulation strategies, which diverted her attention from the negative emotions and made her more diligent and engaged (Han and Xu, 2020).

After the revision, Yuki recalled that the feedback on this writing was still negative, and she felt very guilty. She was still confused about the content of the feedback: “I do not know if I can still write questions when I write papers in the future.” (Oral report) But Yuki said that she would not ask the teacher for advice during class breaks for fear of wasting the teacher’s time and the teacher having a bad impression of her (oral report and class observation), even though feedback is a process in which learners should actively seek, generate, process, and use feedback to apply new knowledge in current or subsequent writing tasks (Teng and Ma, 2024). What is more, growing up in the East Asian cultural circle made her not want her classmates to see her competitive spirit. Feeling guilty, Yuki said that there was no effective way to adjust, and she could only use situation-oriented strategies to avoid feedback sessions that led to her negative emotions. “I can only say do not think about it for now, put it aside, and look at it later.” (Oral report).

#### An adaptive state: groping in the collision of positive and negative emotions in the second and third feedback sessions

After the first feedback session, Yuki had a preliminary understanding of the basic methods of summary writing and had reasonable expectations about academic outcomes and possible writing problems (the third oral report). Therefore, the activation of anxiety arousal before and after the second and third feedback sessions gradually deactivated.

[Second interview].

“I still look at the results after the feedback: 87. The score is higher than last time and also above 85. I feel relieved. The lower the score, the more upset I will be.”

Grades, the result of the writing activity, and the extrinsic value of the writing (Pekrun, 2006) were the most emotionally evocative feedback for Yuki. 85 was the watershed of the normal distribution of grades in her university and also the watershed of her positive and negative emotions. For grades that improved and were above 85, she presented the academic activity emotion: tranquility. Yuki also received positive comments in the second and third feedback sessions, which made her believe that she could make progress as long as she worked hard (second interview). It could be said that grades can pull her from the bottom of the negative-activating emotional experience to the positive-neutral emotional experience, but the teacher’s positive and friendly encouragement can soar her emotions to the positive-activating state, which has a powerful reshaping effect on Yuki’s confidence.

However, there was still confusion in the two writing feedback sessions, and the feedback in class was still good medicine to remove the confusion: it was replaced by emotions such as trust in the teacher or gladness to have “another example to refer to for the next writing.” “The teacher’s encouragement and attitude gave me the motivation to keep working hard” (second interview). As a result, Yuki focused on revising and gradually felt more confident about academic writing. By the third writing, she had basically mastered the writing skills and could estimate the gains and losses of the scores in writing before receiving the feedback (third oral report). In the interview after the last feedback, she used “a little” to describe all her emotional experiences, e.g., “a little anxious.” The accumulation of writing knowledge and skills led Yuki to be full of hope for future writing.

### Sala: academic emotions and regulation strategies in happy growth

Sala is from Thailand. She started learning Chinese because her idol was a Chinese singer. Sala planned to work in a media company in China and write copy to promote the idol’s career, so she put a lot of emphasis on her writing skills. Sala did not care much about her academic grades, believing that as long as she learned and did her best in every course and assignment, she would be fine. She looked forward to receiving feedback on her writing and strongly believed that feedback could help her improve her writing logic and language. Compared to Yuki, Sala’s Chinese writing level was lower, but she made three times more progress in her writing grades, and her emotions were relatively stable. The key times of emotional change and the changes in Sala’s academic emotions are shown in Table 8.

**Table 8:** Sala’s academic emotions to the three feedback sessions and regulation strategies.

		Before receiving	Feedback receiving	Feedback reading	Oral feedback in class	After revision
F1	Academic emotions	Anxiety Expectancy	Tranquility	Confusion expectancy	Satisfaction	Anxiety Relief
	Valence-activation	Negative-deactivating Positive-neutral	Neutral-neutral	Negative-neutral Positive-activating	Positive-activating	Negative-deactivating Positive-neutral
	Strategies	Emotion-oriented regulation				
F2	Academic emotions	Anxiety	Glad	Tranquility	Trust	Tranquility
	Valence-activation	Negative-deactivating	Positive-activating	Neutral-neutral	Positive-activating	Neutral-neutral
F3	Academic emotions	Tranquility	Glad	Curiosity	Tranquility	Hope
	Valence-activation	Neutral-neutral	Positive-activating	Positive-activating	Neutral-neutral	Positive-activating
	Strategies	Appraisal-oriented regulation				

#### A steady start: acquiring knowledge in tranquility in the first feedback session

Sala’s overall assessment of her emotions was “very stable, not particularly happy or unhappy” (third oral report), and the analysis results also showed that this was indeed her emotional experience during the three feedback sessions. After the first summary writing, Sala said that the teacher’s feedback was more important to her than the score. She valued her writing level and academic outcomes and had only a little anxiety before receiving it.

[First oral report].

“I am a bit anxious about the score. I tried my best, and any score was fine. 70–80 is my level of writing. Above all, I want to know what I need to improve.”

After receiving the feedback, Sala felt that “it was fine, I did not feel happy or unhappy, the score of 79 was in line with what I expected of myself” (first interview). The academic results that were in line with expectations made Sala feel calm. Later, when reading the feedback, Sala, like Yuki, had difficulty reading because of the revision mode of the document, which caused confusion. She cautiously read the specific content of the feedback and gradually got used to the way of looking at the feedback:

[First interview].

“I found that I had grasped the wrong key points, and some expressions were too abstract, and the language was not academic enough. I paid more attention to how I could improve my writing skills, so I was very pleased to see so many revisions.”

Acquiring new writing methods from feedback was the most meaningful and valuable thing for Sala. She was satisfied with the new knowledge she had learned, although the confusion still existed. During the interview, Sala’s arms were relaxed and naturally placed on her legs. Her high-pitched voice and constant laughter reflected her positive emotions (Han and Hyland, 2019). Later, the confusion was resolved during the feedback session in class. Sala was looking forward to using new methods in her future writing:

[First interview].

“I did not know that breaking it down into smaller paragraphs would make it clearer until the teacher explained it to me in class. Wow, I can write like that next time.”

Sala then devoted herself to revision and asked Audrey for advice after class on the new confusion. However, the one-on-one oral feedback session triggered Sala’s complex emotional experiences: at first, she was worried about the poor communication with the teacher and felt slightly anxious (she spoke faster and covered her mouth to chuckle when she talked about this), then she began to watch the idol’s inspirational videos to encourage herself and made up her mind to ask the teacher for advice. The subsequent communication with the teacher aroused Sala’s relief and prevented her from being hit: “Even if I blurted out two words, she could understand what I was talking about… I was much clearer after asking her” (first oral report). Moreover, the feedback session in class improved Sala’s self-efficacy and motivated her to invest in the learning process. Sala used an emotion-oriented strategy, that is, watching videos to motivate herself and distract her feelings of anxiety, and finally achieved successful revision (Oxford, 2016).

#### A joyful state: growing rapidly under the infiltration of positive emotions in the second and third feedback sessions

Before receiving F2 and F3, Sala had already noticed her progress in writing while writing, so her emotions gradually became tranquil. She began to receive positive comments: the structure was very clear, and the language was in line with the norm (second feedback text). “I was very glad after reading the comments, and I found that the teacher praised me! “(oral report). Sala’s writing scores increased in both the second and third feedback sessions; the scores were 80 and 88, respectively. However, she believes that the teacher’s comments have the greatest impact on her emotions, much more so than the score itself. Clearly, the content of positive feedback increases students’ self-regulated learning and confidence in the learning task (Mirka and Kirsi, 2019), which in turn generates more positive emotions (Pekrun, 2006).

F3 also evoked Sala’s cognitive emotion of curiosity: “The teacher marked that this sentence was wrong. I also looked through my other writings to see if there was the same mistake, and sure enough, there was” (third interview). The mistakes marked by Audrey reminded Sala of similar problems in previous papers. Feedback can help her transfer language knowledge to multiple studies.

F2 in class still aroused Sala’s trust in the teacher. She believed that it could effectively sort out writing errors and consolidate writing knowledge based on written feedback. Sala gradually adapted to and got used to the revision, and her emotional experience became tranquil. After the last revision, according to the feedback, Sala found that she was more familiar with academic writing and had made significant progress (class observation). At the same time, Sala found that she had become more manageable with writing in other courses: “There was a class that asked us to write a book report. I introduced the book in four points (research background, research questions, research methods, and evaluation) according to the teacher’s feedback, and the quality was very good” (second interview and oral report). The use of evaluative strategies made Sala feel hopeful about the feedback.

## Discussion

Informed by the analytical framework of academic emotions (Pekrun and Linnenbrink-Garcia, 2012) and emotions regulation strategies (Pekrun, 2006), this study examined the emotional experiences and emotional regulations of two Chinese academic writing novices in the context of three writing feedback sessions in one semester. The data showed that before and after receiving feedback, the two CFL students experienced as many as 16 kinds of achievement emotions, cognitive emotions, and social emotions with different levels of valence and arousal, and they could use a variety of strategies to regulate emotions. These emotional experiences were intertwined, fluctuating, and evolving along the timeline of “before receiving feedback, just after receiving feedback, and during in-class feedback in 1 to 5 days after revision” (Han and Xu, 2020). These changes occurred within 1–5 days after each feedback session and throughout the semester across three feedback cycles.

The above research results further confirm the framework proposed by Pekrun (2006) and Pekrun and Linnenbrink-Garcia (2012) for analyzing academic emotions and their regulatory strategies. In addition, by incorporating emotions such as conflict, novelty, achievability, relief, and anticipation, the study suggests that the taxonomy of academic emotions (Pekrun and Linnenbrink-Garcia, 2012) needs to be expanded and adapted for feedback sessions. The academic emotions aroused by feedback underwent complex changes, not merely shifting from negative to positive. Emotions could be transient or persist throughout the entire feedback processing cycle (Han and Xu, 2020; Kikuchi and Lake, 2021). This result differs from previous studies, which state that feedback always triggers negative emotions (Truscott, 1996). However, the emotions of the two CFL students gradually became positive in the three feedback sessions. Even Yuki, who often experienced anxiety at the beginning, gradually felt tranquil and grateful in the second and third feedback sessions. The same discrete emotion varied along the activation. For example, before receiving feedback, both students felt anxiety, but the activation was different: Yuki was activating, while Sala was only deactivating. This finding forced us to adjust the taxonomy of emotions as seen in previous studies (Pekrun and Linnenbrink-Garcia, 2012). Emotional reactions with the same valence but different activation levels had an impact on students’ motivation and learning effects (Pekrun et al., 2002; Geng and Yu, 2024). Yuki was trapped in activating anxiety and had difficulty engaging in the subsequent modification after receiving feedback. At the same time, Sala quickly entered the learning state and immediately took corrective actions, indicating that learners with stronger psychological resilience will actively deal with negative emotions, think about the problems presented in the feedback, and seek value from it (Tugade and Fredrickson, 2004).

We found that compared to written feedback, oral feedback in class was always a good medicine to relieve their nervousness and anxiety, which can help them understand the content of feedback and regulate their emotions. The results also showed that the discrete emotions aroused by the two learners at different stages of feedback showed similar tendencies. Written feedback from the teacher may be more likely to evoke negative emotions in learners. In the learners’ process of dealing with written feedback, firstly, in terms of prospective outcome emotions, both students exhibited negative emotions before receiving feedback and felt nervous and anxious about the score and the content of the feedback because feedback is usually regarded by learners as an evaluation of the quality of academic writing (Mahfoodh, 2017; Geng and Yu, 2024). Second, in terms of activity emotions, both learners had different negative emotions, such as anxiety and confusion, in the first feedback (F1) session when they were engaged in the feedback activities (receiving and reading the feedback); fortunately, these negative emotions did not affect their revision and reflection on their writing too much, and their emotions gradually tended to be neutral and positive in the following two feedback sessions. Finally, after revising their writing according to the feedback, both of them showed retrospective outcome emotions such as tranquility and hope as the number of feedback sessions increased. It can be seen that engagement with feedback and new knowledge may be able to smooth out learners’ negative emotions to a great extent and increase their happiness and satisfaction with learning.

In addition to academic achievement emotions, Yuki and Sala also experienced epistemic emotions such as novelty or confusion when they first encountered feedback on Word’s “document revision mode.” They regarded the feedback as a manifestation of the teacher’s concern for their learning and the teacher’s serious work (Lee, 2008a,b; Han and Xu, 2020), so the feedback also awakened their social emotions, such as gratitude and relief. These social interactions with the stakeholders also helped novice researchers enter the academic discourse community (Geng and Yu, 2024).

With the increase in the frequency of feedback sessions, the emotions of students gradually tended toward positivity. In addition to their gradual adaptation to the feedback content and improvement in writing skills, they also benefited from the appropriate use of their emotion regulation strategies. Both learners used emotion-oriented strategies. For example, Sala adopted an emotion-oriented regulation strategy of watching idol videos repeatedly to relax and reduce negative emotions when faced with anxiety during the one-on-one feedback session with the teacher. The idol was both a motivation for her to learn Chinese and could encourage her to bravely ask the teacher for advice. It can be seen that positive emotions can reduce negative emotions to a certain extent and help learners reduce the destructiveness of negative emotions (Oxford, 2016; Han and Xu, 2020; Gu et al., 2022). However, when Yuki, who used the situation-oriented strategy, faced feedback that put her in a negative mood, she chose to avoid the feedback text that triggered her negative emotions. As she grew up in the implicit and introverted culture of East Asia, she was concerned about revealing her true self to her classmates. Although she still had doubts and confusion about the feedback and was under great pressure from her GPA throughout the year, she was not encouraged to seek support and solve problems from teachers or peers. In comparison, the appraisal-oriented strategy was more effective in regulating emotions and had a far-reaching impact. Sala used the appraisal-oriented strategy, and her positive attitude toward the role of feedback (positive value appraisal) and confidence in her progress (positive control appraisal) led her to pay more attention to feedback (Pekrun, 2006) and increased her satisfaction with revision. Finally, she was full of hope for future academic writing.

In addition, previous studies have suggested that learners with low language proficiency are more likely to receive more feedback and, therefore, may experience more negative emotions with a higher degree of activation (Hyland, 1998). However, this study found that this hypothesis may not reflect the actual experience of CFL students: Yuki, whose language proficiency and writing scores have been among the top all along, has mainly negative emotional experiences, while Sala, whose language proficiency and scores were initially low, is mostly in a positive mood. This finding suggests that L2 learners’ emotional experience in feedback is not solely influenced by language level and feedback content. To explore the factors that affect learners’ emotional experience, it is necessary to combine a wider range of situational factors (Bruton, 2009, 2010). First, different forms of teacher feedback have different effects on learners’ emotions. Written feedback is usually more likely to arouse learners’ negative emotions. Compared with the single-modal (visual) feedback input in writing, the auditory and visual multimodal feedback of teachers’ tone, expression, and actions in oral feedback were more helpful in solving learners’ confusion and alleviating their negative emotions. After receiving one-on-one feedback or feedback in class, the two CFL students mostly turned to positive emotions, which proved to be a good corrective effect of oral feedback.

Second, the students’ perceptions of the controllability of writing outcomes could also evoke their different emotional experiences. For example, the lower score in the first feedback session made Yuki think that her writing was a failure, thus lowering her subjective control assessment of the writing task, and negative emotions were generated as a result. The improvement of her writing scores and the appearance of positive comments in the following F2 and F3 helped her realize her ability to control the outcomes of the academic activities, and her emotions gradually became positive. Finally, this study also found that paying attention to the internal value of feedback could help the students experience more positive academic emotions. Both CFL students had a high level of engagement, while their subjective value appraisal of feedback was significantly different: Sala attached importance to internal value and believed that revising feedback could acquire writing knowledge. She often had emotional experiences of gladness and achievement from mastering writing skills. Therefore, she paid more attention to feedback regardless of whether she received good grades or not. However, Yuki’s high investment in revising feedback was mostly driven by the desire for a high GPA (external value) and viewed feedback itself as a tool to achieve academic achievement (Papi, 2010). Therefore, when academic outcomes that did not meet expectations appeared, Yuki could only feel a lot of negative emotions, regardless of whether she had mastered writing skills during the revision feedback process.

The findings further support the view that emotion research from the perspective of positive psychology needs to be contextualized (Han and Xu, 2020). Judging from the academic achievements of the three summary writing tasks, both Yuki and Sala, the high-level and low-level Chinese writers, respectively, undoubtedly made progress. However, Sala, who “did not care much about the scores,” made more remarkable progress. Yuki kept her eyes fixed on the goal and just kept going and going. Therefore, it is no wonder that the crazy attacks of tiredness and anxiety came. Guided by the goal of pursuing scores and GPA, Yuki actively conducts self-discipline and self-management and gradually grows into a “calculable person” (Foucault, 2012). The findings of this study support the control-value theory (Pekrun, 2006). Moderate self-imposed pressure is a condition that can promote deep learning. However, excessive self-imposed pressure may heighten docility, devalue creativity, and thus hinder the occurrence of real learning (Lin and Lin, 2023). At this time, knowledge is overshadowed by the practical, insignificant, and dull, and the time for students to develop critical thinking and exploratory thinking is immediately compressed indefinitely. Eventually, the emphasis on the learning style for the purpose of assessment and the excessive self-imposed harsh requirements for emotions lead to a decline in learners’ motivation and morale and induce learners’ hopelessness and anxiety toward academic and learning activities in terms of emotions (Lin and Lin, 2023).

Sala was eager to write knowledge and enjoyed the learning process. She not only has a positive value appraisal of summary writing and its feedback but also has sufficient confidence to make progress with the help of teachers’ feedback and looks forward to positive future academic achievements. She walked, enjoyed the flowers, and grew strongly accompanied by happiness and tranquility. The accumulation of state emotions that occur frequently will form trait emotions (Pekrun et al., 2011). If students can effectively regulate their emotions and often experience positive academic emotions, they are more likely to build positive personality traits, thereby gaining more positive psychological resources to cope with challenges and ultimately achieve a double harvest of academic achievement and happiness (Han and Xu, 2020).

## Conclusion

This study provides empirical data on how to improve happiness in the process of learning Chinese second language academic writing from the perspective of positive psychology. Compared with previous studies on feedback texts and feedback emotions of master’s and doctoral students, this study uses a case narrative method to deeply describe the two CFL students’ academic emotional experiences’ changes and emotion regulation strategies of three feedback sessions in one semester, revealing the dynamics, richness, and complexity of academic emotions from multiple dimensions such as discrete emotions, activation, valence, and object focus. The results indicate that both positive and negative emotions have different promoting effects on the academic achievements of CFL students, helping them engage in writing learning and strive for academic improvement. Meanwhile, CFL students can also exert positive personality traits, regulate emotions, enhance resilience and well-being, and re-engage in the problem-solving process (Gross, 2015; Han and Xu, 2020; Oxford, 2016).

However, this study also has the following limitations. First, we only used writing ratings as measures of academic achievement. Future research could use more sophisticated measurement methods, such as lexical and syntactic complexity and accuracy, to gain a better understanding of the links between emotions and L2 writing achievement. Second, it is difficult to obtain and analyze CFL students’ emotional reactions to feedback through interviews and oral reports, and there may be problems such as inaccurate language expression, inaccurate emotion perception, and memory of L2 students.

Future studies could incorporate multiple measures to assess processing, such as combining traditional methods with tools such as eye-tracking, to gain a deeper understanding of feedback engagement. Additionally, the sample size and research environment of this study may limit the generalizability of the results. Despite these limitations, this study highlights the importance of exploring cultural and linguistic variability to enrich the application of positive psychology in language learning.

Future research could address the following aspects. For example, examining potential differences in learners’ values, expectations, and attitudes toward teacher feedback across different cultural backgrounds, analyzing how the linguistic characteristics and learning challenges of learners’ native languages and the target language (Chinese) influence emotional experiences. Such investigations would help ensure sample diversity and representativeness, particularly in terms of cultural background, thereby broadening the scope and applicability of findings in this field.

This study also sheds light on how academic writing teachers can provide feedback in a more acceptable and understandable way and how novice writers can use feedback to improve their academic writing skills. First, teachers should understand the complexity of learners’ emotions rather than assuming that feedback will inevitably trigger negative emotions that interfere with L2 writing learning. They should recognize that even negative feedback can trigger positive emotions and that learners can self-regulate negative emotions. Therefore, teachers need to explore how to use emotions in writing feedback, such as guiding students to express and reflect on emotions and increasing their awareness of the value of academic emotions.

Second, they could provide clear support for emotional issues triggered by academic writing feedback to help second-language learners regulate their emotions (Goetz et al., 2006). For example, they could help students establish a subjective control appraisal centered on self-agency, fostering a belief that they can improve their writing skills by actively engaging with feedback (Han and Xu, 2020). Teachers could also guide learners to make positive subjective value judgments about the importance of feedback, revisions, and writing tasks, helping students to regulate their emotions, and build psychological resilience, enhance their ability to benefit from feedback.

Finally, teachers also need to pay attention to the following points when giving feedback: First, they should provide guidance to students on how to review feedback so that they are not confused about the revision mode; second, they should pay attention to the effectiveness of feedback comments and strike a balance between positive and negative comments. This study found that negative feedback does not always evoke negative emotions while helping students to identify their own problems and make targeted improvements in future writing; positive feedback helps students to improve their self-agency in writing, recognize their own strengths, and develop them further. However, teachers generally focus on students’ language or structural content problems, use negative comments, and ignore positive comments when giving feedback, which is not conducive to building students’ confidence and enthusiasm for writing. If teachers strike a balance between correction, praise, and encouragement, would could effectively stimulate students’ interest in writing and foster a positive cycle of academic writing and feedback.

## Data Availability

The original contributions presented in the study are included in the article/Supplementary material, further inquiries can be directed to the corresponding author.
