# Supplementary material for: Self-imposed pressure or joyful learning: emotions of Chinese as a foreign language learners in feedback on academic writing
Source: Front Psychol. 2025 Jan 7;15:1463488. doi: 10.3389/fpsyg.2024.1463488 (PMC11771137; doi:10.3389/fpsyg.2024.1463488)
Supplement: Supplementary file 2 [file Data_Sheet_2.pdf]

Self-imposed pressure or joyful learning: emotions of Chinese as a foreign language learners in feedback on academic writing

APPENDIX A: Guide for the interview

1. Please tell us some basic information about yourself, including your age, nationality, etc.
2. Have you written Chinese academic writing before? What are your experiences? How has the teacher's feedback helped you?
3. What happened and how did you feel emotionally before receiving feedback?
4. What happened and how did you feel emotionally in the process of receiving feedback?
5. What happened and how did you feel emotionally when you revised your manuscript according to the feedback?
6. How did you make use of the feedback in your revisions?
7. What happened and how did you feel emotionally after receiving feedback?
8. What happened and how did you feel emotionally When the teacher gave you the oral feedback on class?
9. Which part of the feedback (for example, the teacher's comments, the score) has the greatest impact on your emotions?
10. How did you manage your emotions after receiving the feedback?
11. Could you use an example to illustrate how the feedback affect your writing?

APPENDIX B: Codes and explanations of discrete feedback-aroused emotions (adapted from Han & Hyland, 2019)

Discrete emotions	Description
Conflict	The feeling of being torn because you don't know exactly which way is better for you
Anxiety	Being worried about and fearful for the failure anticipated to occur
Hopelessness	Being pessimistic about future success
Guilt	The feelings that the failure was due to one's specific, local action and corrective action could be made toward reparation
Novelty	Feeling strange and novel because of something you have never seen or experienced before
Confusion	A feeling of not understanding or knowing what to do with the feedback
Tranquility	Being neutral in terms of valence and activation
Gratitude	The feeling that the student wanted to thank the teacher for her good will and effort
Relief	The students feel at ease because the things they were worried about did not happen or had good results.
Achievability	The feeling when desires and reality are balanced
Glad	The feeling of happy and joyful
Trust	The feeling of having a strong belief in the quality of feedback provided by the teacher or in the teacher's good will in providing feedback
Hope	The feeling of anticipating future success
Expectancy	The feeling of longing and yearning for something unknown in the future.
Curiosity	Becoming interested in understanding the errors marked with WCF
Satisfaction	The feeling of having a psychological state of pleasure and approval towards the feedback.
